# Innovation in education. Commentary: Teaching statistics using dance and movement and a case for neuroscience in mathematics education

**DOI:** 10.3389/fpsyg.2016.00694

**Published:** 2016-05-13

**Authors:** Carl Senior

**Affiliations:** Department of Psychology, School of Life and Health Sciences, Aston UniversityBirmingham, UK

**Keywords:** mathematics, dance cognition, numerical cognition, pedagogy, innovation

Mathematical expertise is central to our understanding of science, drives innovation throughout all aspects of society and ultimately facilitates the economy of nations (OECD, 2010). There is little doubt as to its importance and as such it is taught to varying degrees of success across nearly all educational systems (Kaiser and Sriraman, 2006). Yet certain cultures regularly still show an advantage over others in developing mathematical expertise (Tcheang, 2014). Take the example of the competencies revealed in Asian cultures that may be due, in part, to the almost ubiquitous use of the abaci throughout Schools in Asia (Brinkworth, 1997). Regular training on the abacus during the formative school years has been shown to recruit cortical regions that are implicated in the computation of visuospatial processing in the service of mathematical computations (Hatano et al., 1977; Stigler, 1984; Ku et al., 2012). Such evidence highlights the effectiveness of certain interventions within school level educational programmes that drive competencies in later life but also shows that interventions do have an impact at the cortical level. What is not known is whether there are other such naturalistic interventions that could be developed to improve mathematical competency.

There are obvious advantages to the identification of educational programmes that align themselves to naturalistic behaviors. Notwithstanding the economic benefits of designing student activities that would also occur outside of the classroom there is a clear advantage to the effective delivery of activities that develop a transferable skillset that will eventually benefit the world of work (Bridges, 1993). The alignment of didactic practice to naturalistic social behaviors has been termed *mise-en-place*. This is a phrase taken from professional catering referring to the manner in which a chef ensures that a workstation is appropriately organized to take advantage of his natural movements to ensure an efficient service (Skolnick-Weisberg et al., 2014). Contemporary educationalists have adopted the phrase to describe the benefits realized by reshaping the physical learning environment so that it supports the execution of more socially relevant and naturalistic behaviors (Skolnick-Weisberg et al., 2014).

With regards to mathematic competencies the adoption of a *mise-en-place* type approach has already been shown to recruit additional cortical regions in the computation of mathematical problems (Grabner et al., 2007; De Smedt et al., 2011). Indeed the interaction between this type of problem solving and education at the level of cortical systems has recently been highlighted by Susac and Braeutigam (2014) who also argue that effective policy and practice can and indeed should be informed by this literature. They go on to argue that application of neuroimaging technology could be used to identify specific developmental stages of key cortical regions and educational practice should be designed around these stages with specific interventions being delivered at various stages of our developmental and educational trajectory. Such an approach is inherently provocative however prior to realizing a developmentally informed educational practice one must consider mathematical competency in its natural social ecology—that is maths in the real world and furthermore how an understanding of mathematics can be informed by the way we move throughout the natural world.

The relationship between mathematical competency and natural movement is, perhaps surprisingly, not contentious. Babies readily explore the world around them by moving objects around the room and seeing if they fit into smaller spaces etc. Such fundamental behaviors are essential for understanding the cardinality of the world and lays the foundation for an understanding of ordinal numerosity (Dantzig, 2007). In older adults physical movement also plays a role in supporting mathematical proficiency and need not be complex in order to facilitate understanding. Taking part in a short activity period of basic movement skills, e.g., jumping jacks etc. leads to a significant improvement in mathematics (Jaakkola et al., 2015). Movement skills leads to the ability encode spatial relationships in the world around you and this visuospatial skill predicts mathematical competency (Dehaene et al., 1999). Further the fact that deficits in visuospatial abilities also leads to deficit in mathematical proficiency in various psychiatric disorders suggests a comorbid dysfunction (Nelson and Shankman, 2016) and adds convergent support for the presence of overlapping cognitive processes. While an understanding of the co-opted nature between movement and mathematical proficiency has been used in the design of effective pedagogy (Nemirovsky et al., 2013; Carlson et al., 2014). Here it would seem that the study of movement and the underlying visuospatial cognitions and maths has an academic pedigree.

The effectiveness of physical activity in driving learning has previously been seen with the use of dance (e.g., Bruner, 1966; Laban, 1966). This early work is significant when one considers the importance of dance, which is a deliberate physical activity, in driving mathematical proficiency (Werner, 2001). Yet despite its deliberate nature some theorists argue that dance played an important role in our evolutionary past and allowed us to communicate various concepts in a nonverbal and naturalistic fashion (Hanna, 1979; Karpati et al., 2015). This naturalistic form of communication is a universal feature in human culture and may have facilitated the development of more complex cognitive processes such as language and even general intelligence (Mithen, 2005). Here it is clear that concepts such as geometry and symmetry can be readily mapped to the body shapes that would be practiced with Dance and that these naturalistic physical movements that are practiced in the dance studio can also be mapped to the understating of mathematical concepts (Tahta, 1989; Belcastro and Schaffer, 2011). Dance may in fact be the *mis-en-place* type intervention that could facilitate mathematical competence (Irving, 2015)^1^.

Even taking this evidence in hand there are still a number of unanswered questions that need to be addressed before a fully naturalistic approach to the teaching of mathematics can be formulated. To what extent does dance movement facilitate mathematical competency in later life? Would such training at the undergraduate level have a similar effect on mathematical proficiency as it does in younger leaners? What is the effect of improvised dance compared to codified dance forms ? Also, does it have a moderating effect or more of a mediating effect on the facilitation of existing knowledge? Furthermore does dance training have any impact on the developmental knowledge on specific mathematical competencies or does it play a general facilitative role? Despite the emerging body of research examining the relationship between dance and mathematics these questions remain unanswered.

## Conflict of interest statement

The author declares that the research was conducted in the absence of any commercial or financial relationships that could be construed as a potential conflict of interest.

## Footnotes

^1^Indeed, contemporary educationalists have already developed unique dance programmes for early stage learners (e.g., www.mathsdance.com). Such programmes not only serve to facilitate the foundation for mathematical proficiency in later years but also to further consolidate the importance of codified dance forms e.g., classical ballet or contemporary and modern dance etc) as a means to deliver effective learning in this area (see Ornes, 2013 for a further discussion).
